# Supplementary material for: CryoSIM: super-resolution 3D structured illumination cryogenic fluorescence microscopy for correlated ultrastructural imaging
Source: Optica. 2020 Jul 13;7(7):802–12. doi: 10.1364/OPTICA.393203 (PMC8262592; doi:10.1364/OPTICA.393203)
Supplement: Supplementary file 1 [file optica-7-7-802-s001.pdf]

Supplemental Document

optica

CryoSIM: super-resolution 3D structured illumination cryogenic fluorescence microscopy for correlated ultrastructural imaging: supplement

MICHAEL A. PHILLIPS,[1,2,3] MARIA HARKIOLAKI,[3] DAVID MIGUEL SUSANO PINTO,[1] RICHARD M. PARTON,[1] ANA PALANCA,[4,5] MANUEL GARCIA-MORENO,[5] ILIAS KOUNATIDIS,[3] JOHN W. SEDAT,[6] DAVID I. STUART,[2,3] ALFREDO CASTELLO,[5] MARTIN J. BOOTH,[7] ILAN DAVIS,[1,8] AND IAN M. DOBBIE[1,*]

[1] *Micron Advanced Bio-imaging Unit, Department of Biochemistry, University of Oxford, South Parks Road, Oxford OX1 3QU, UK*
[2] *STRUBI, Division of Structural Biology, Wellcome Centre for Human Genetics, Old Road Campus, Roosevelt Drive, Oxford OX3 7BN, UK*
[3] *Diamond Light Source, Harwell Science & Innovation Campus, Didcot, Oxfordshire OX11 0DE, UK*
[4] *Department of Anatomy and Cell Biology, Faculty of Medicine, Universidad de Cantabria, CP39011 Santander, Spain*
[5] *Department of Biochemistry, University of Oxford, South Parks Road, Oxford OX1 3QU, UK*
[6] *Department of Biochemistry and Biophysics, University of California, San Francisco, California 94143, USA*
[7] *Department of Engineering Science, University of Oxford, Parks Road, Oxford OX1 3PJ, UK*
[8] *e-mail: ilan.davis@bioch.ox.ac.uk*
[*] *Corresponding author: ian.dobbie@bioch.ox.ac.uk*

This supplement published with The Optical Society on 13 July 2020 by The Authors under the terms of the Creative Commons Attribution 4.0 License in the format provided by the authors and unedited. Further distribution of this work must maintain attribution to the author(s) and the published article's title, journal citation, and DOI.

Supplement DOI: https://doi.org/10.6084/m9.figshare.12446108

Parent Article DOI: https://doi.org/10.1364/OPTICA.393203

CryoSIM: super-resolution 3D structured illumination cryogenic fluorescence microscopy for correlated ultrastructural imaging: supplement

Michael A. Phillips[1,2,3], **Maria Harkiolaki**[3], **David Miguel Susano Pinto**[1], **Richard M. Parton**[1], **Ana Palanca**[4,5], **Manuel Garcia-Moreno**[5], **Ilias Kounatidis**[3], **John W. Sedat**[6], **David I. Stuart**[2,3], **Alfredo Castello**[5], **Martin J. Booth**[7], **Ilan Davis**[1,@], **and Ian M. Dobbie**[1,*]

[1]*Micron Advanced Bio-imaging Unit, Department of Biochemistry, University of Oxford, South Parks rd, Oxford OX1 3QU, UK*
[2]*STRUBI, Division of Structural Biology, Wellcome Centre for Human Genetics, Old Road Campus, Roosevelt Drive, Oxford, OX3 7BN, UK*
[3]*Diamond Light Source, Harwell Science & Innovation Campus, Didcot, Oxfordshire, OX11 0DE, UK*
[4]*Department of Anatomy and Cell Biology, Faculty of Medicine, Universidad de Cantabria, CP39011 Santander, Spain.*
[5]*Department of Biochemistry, University of Oxford, South Parks rd, Oxford OX1 3QU, UK*
[6]*Department of Biochemistry and Biophysics, University of California, San Francisco, USA*
[7]*Department of Engineering Science, University of Oxford, Parks Road, Oxford,OX1 3PJ, UK*
[@]*Joint corresponding author: ilan.davis@bioch.ox.ac.uk*
[*]*Joint corresponding author: ian.dobbie@bioch.ox.ac.uk*

Compiled May 27, 2020

This document provides supplementary information to "CryoSIM: super-resolution 3D structured illumination cryogenic fluorescence microscopy for correlated ultrastructural imaging," https://doi.org/10.1364/OPTICA.393203.

1. SUPPLEMENTAL FIGURES

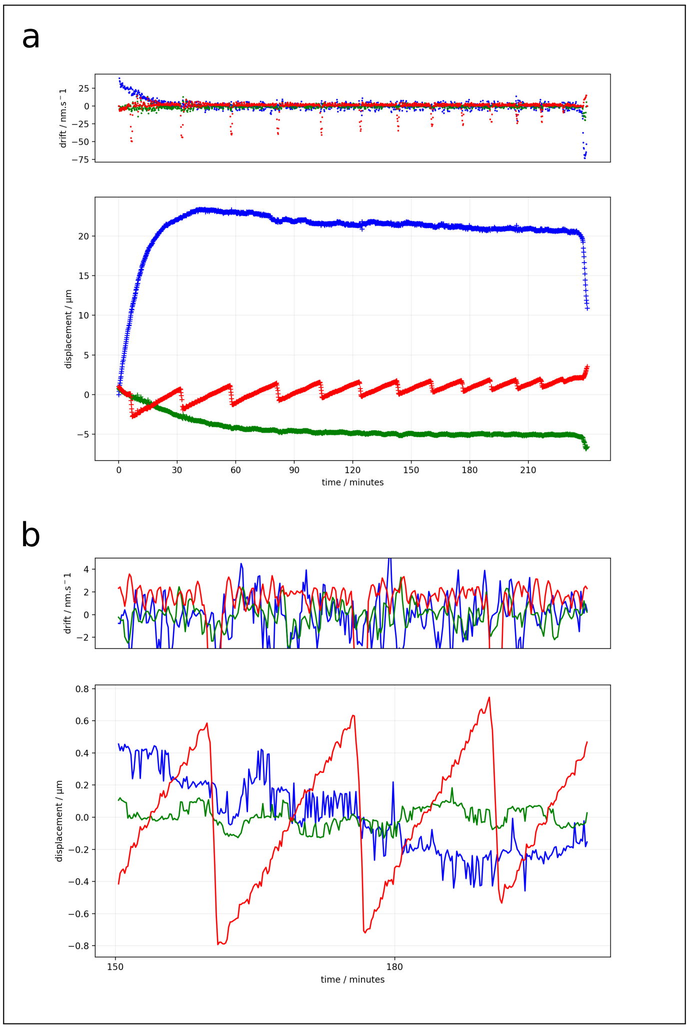

Fig. S1. System drift over time: Drift measured from bead centroids in 3D image stacks. (**a**) Drift rate and total drift over 4 hours in X (red), Y (green) and Z (blue). There are large changes in displacement for the first 30 minutes, especially in Z as the stage cools from room temperature down to cryogenic temperatures. Then there is a cyclic change as the on stage liquid nitrogen dewar is filled rapidly and empties over about 20 minutes. (**b**) The same data plotted from 150-200 minutes. It can be seen that the drift is in nm/s range with the x-axis having about 2 nm/s positive drift between refills. Mean displacements are fairly random about 0.2 μm in z and 0.1 μm in y over a cycle, while x undergoes a repeatable, slow drift of about 1.4 μm.

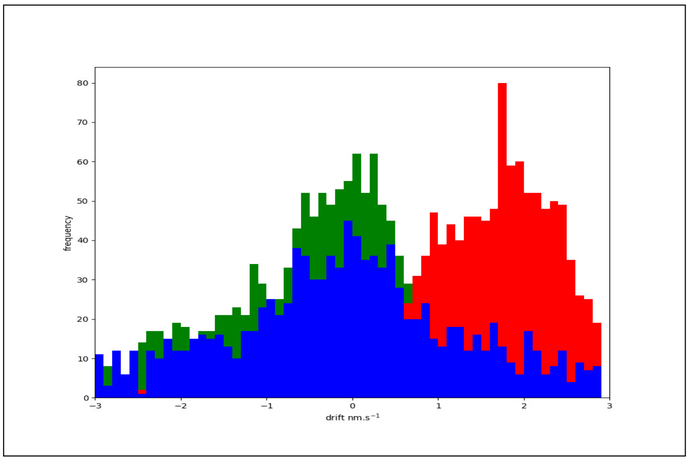

Fig. S2. Histogram of system drift over time: Drift rates in X (red), Y(green), and Z(blue), showing that Y and Z are roughly Gaussian distributions around 0 nm.s^{-1} while the X direction has a distribution around 2 nm.s^{-1} due to the rapid fill and slow emptying of the on stage dewar.

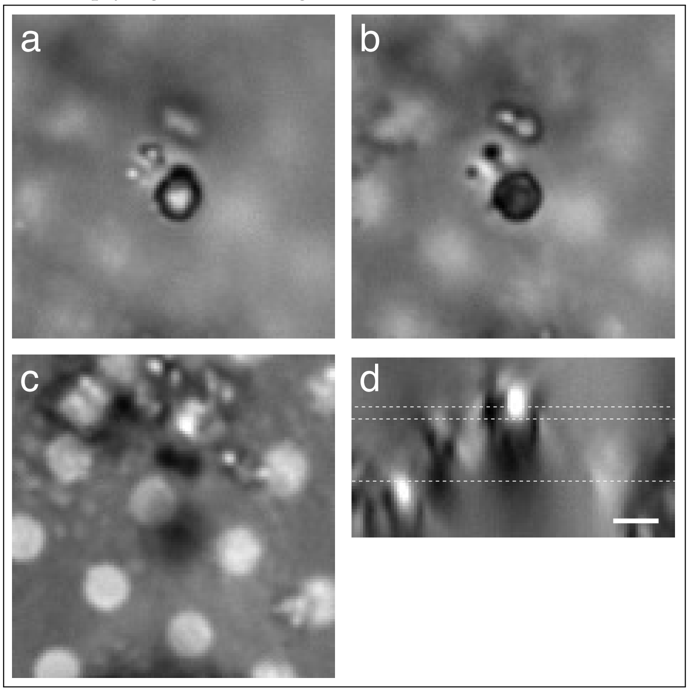

Fig. S3. Measurement of ice thickness above the sample: Transmission light images of plunge frozen U2OS cells. (**a**) An in focus ice crystal on top of the vitreous sample.(**b**) The same region with the focus moved down until the first sample features, 2 black vesicles just next to the ice, are in focus. (**c**) The same region again, with the carbon film supporting the sample in focus. (**d**) A YZ image through the sample at the ice crystal, with the position of the infocus plane on the ice crystal, marked at the top, 2.625 μm above the carbon, the first sample features, marked in the middle, 1.875 μm above the carbon, and the in focus carbon film at the bottom, marked with dashed white lines. The ice thickness must be less than 0.75 μm. Scale bar 2 μm.

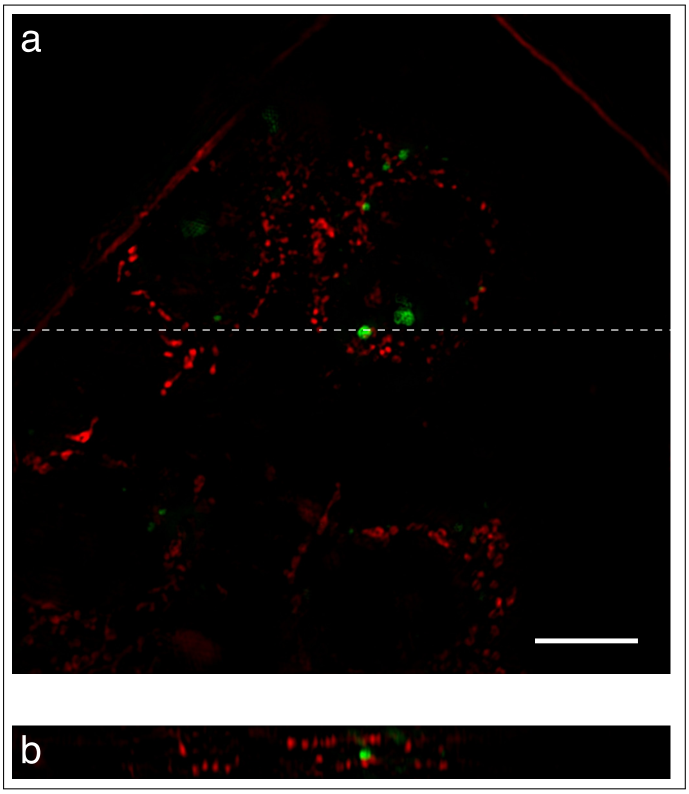

Fig. S4. XY and XZ slices from Fig. 6: HeLa cells expressing mov10-YFP and labelled with MitoTracker Deep Red. (**a**) SIM reconstruction fluorescence image from A maximum intensity projection over 3.125 μm with mov10-YFP in green and MitoTracker Deep Red in red. (**b**) XZ slice though the 3D data stack along the dashed line in **a**. Scale bar 10 μm.

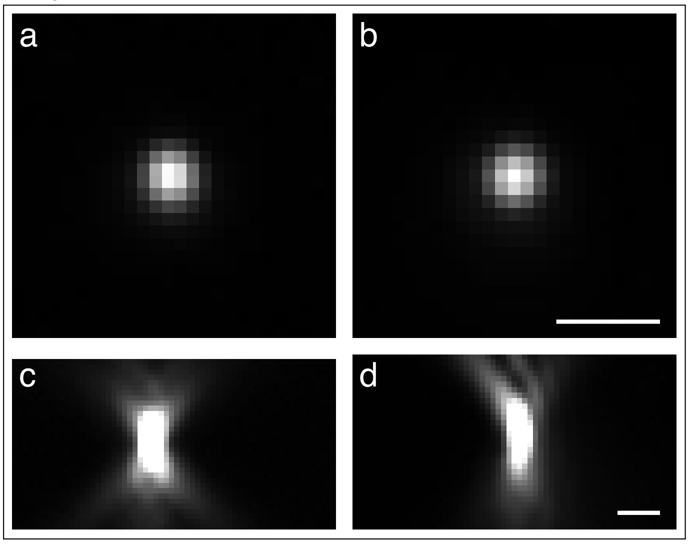

Fig. S5. Room temperature and Cryo PSF: Single 175 nm fluorescent beads illuminated at 488 nm and observed at with a 544/24 nm emission filter. All images are widefield. (**a,b**) in focus XY views at room and cryo temperatures. (**c,d**) XZ slices of the 3D image stack at room and cryo temperatures. These images have a lower maximum display threshold to clearly show the out of focus aberrated point spread function at cryo temperatures in **d**. Scale bars 1 μm.

2. TECHNICAL DRAWINGS FOR CUSTOM PARTS

The CryoSIM instrument is almost entirely constructed from commodity parts. There are a few specialist parts such as the cryo-stage and the custom made dichroic, however these parts are also commercially available. There are a small number of custom made, or adapted pieces. These are involved in the objective lifer mechanism and in cryostage stage mount which includes both precision rails for sliding the sample into and out of the optical path, and a manual coarse focus mechanism. These parts are detailed in at attached technical drawings and CAD files of them are also available on request. The following table has a list of the files and what they relate to.

Table S1. Technical Drawings for custom parts

File	Description
ObjectiveMotion.pdf	Objective lifter mechanism full assembly, and diagrams of custom parts and part modifications required.
LinkamCarriage.pdf	Base plate for stage to run on rails.
ZActuator.pdf	Manual rotation wheel turns lead screw.
Plate.PiezoToLeadscrew.pdf	Plate under the Piezo stage that runs on the lead screw.
Plate.PiezoToCryostage.pdf	Adaptor plate from piezo to mount Linkam stage on.
BushingSpacer.pdf	Spacer to allow dual bushings and prevent the stage twisting and binding on bushings.
DewarCarriage.pdf	Base plate for dewar to run on rails.
RollerPlungerMount.pdf	Mount for a roller plunger to allow reproducible positioning of stages on rails.
