# Supplementary material for: CryoSIM: super-resolution 3D structured illumination cryogenic fluorescence microscopy for correlated ultrastructural imaging
Source: Optica. 2020 Jul 13;7(7):802–12. doi: 10.1364/OPTICA.393203 (PMC8262592; doi:10.1364/OPTICA.393203)
Supplement: Supplementary file 2 [file optica-7-7-802-d001.zip › LinkamCarriage.pdf]

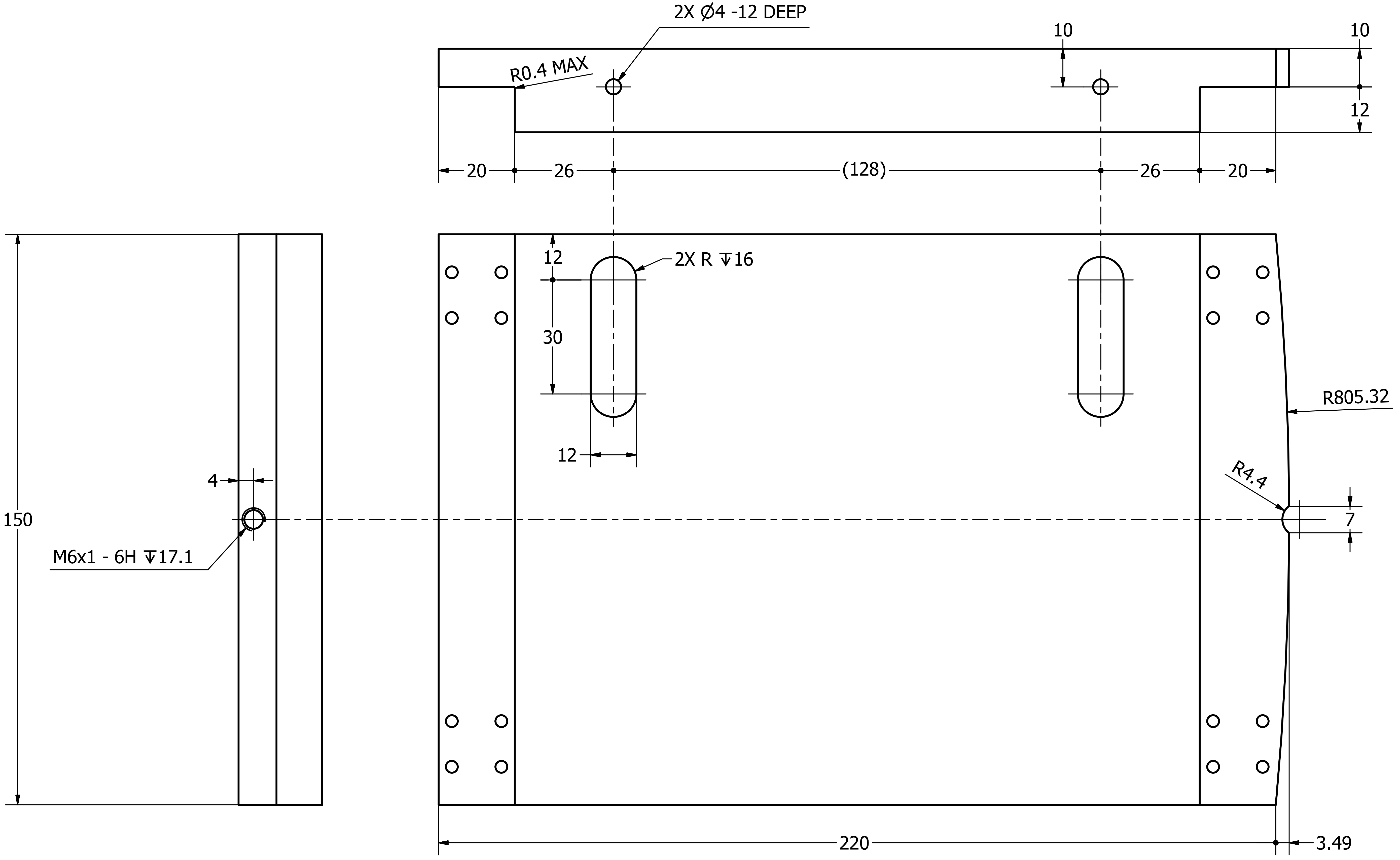

1 OFF, ALUMINIUM

Designed by	Checked by	Approved by	Date	Date
map				14/08/2018

3rd ANGLE PROJECTION

Micron Oxford

LinkamCarriage

Edition

Sheet 1 / 2

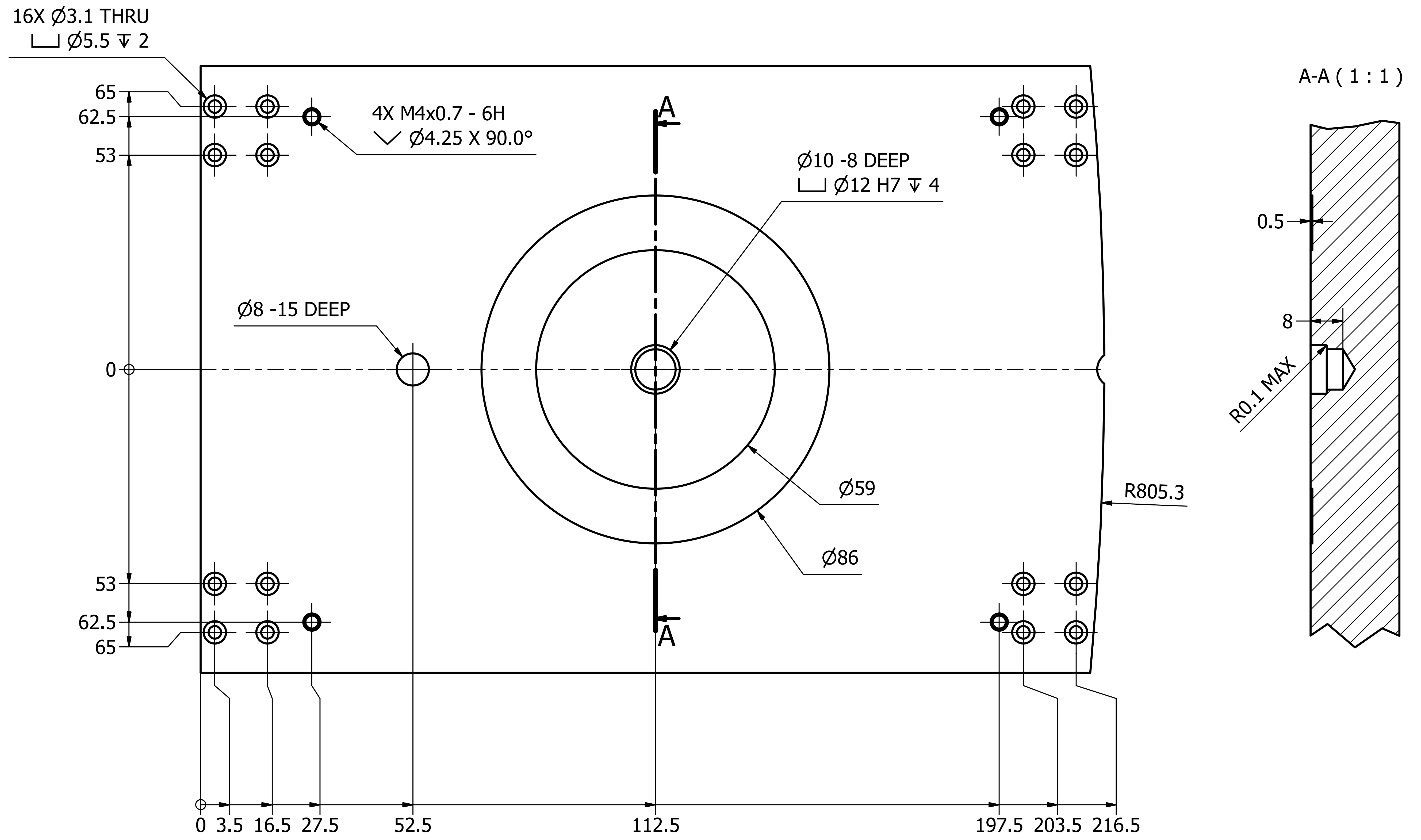

Designed by: map
Checked by:
Approved by:
Date:
Date: 14/08/2018

3rd ANGLE PROJECTION

Micron Oxford

LinkamCarriage

Edition:
Sheet: 2 / 2
