# Supplementary material for: CryoSIM: super-resolution 3D structured illumination cryogenic fluorescence microscopy for correlated ultrastructural imaging
Source: Optica. 2020 Jul 13;7(7):802–12. doi: 10.1364/OPTICA.393203 (PMC8262592; doi:10.1364/OPTICA.393203)
Supplement: Supplementary file 2 [file optica-7-7-802-d001.zip › ObjectiveMotion.pdf]

mirror mount (1 : 1)

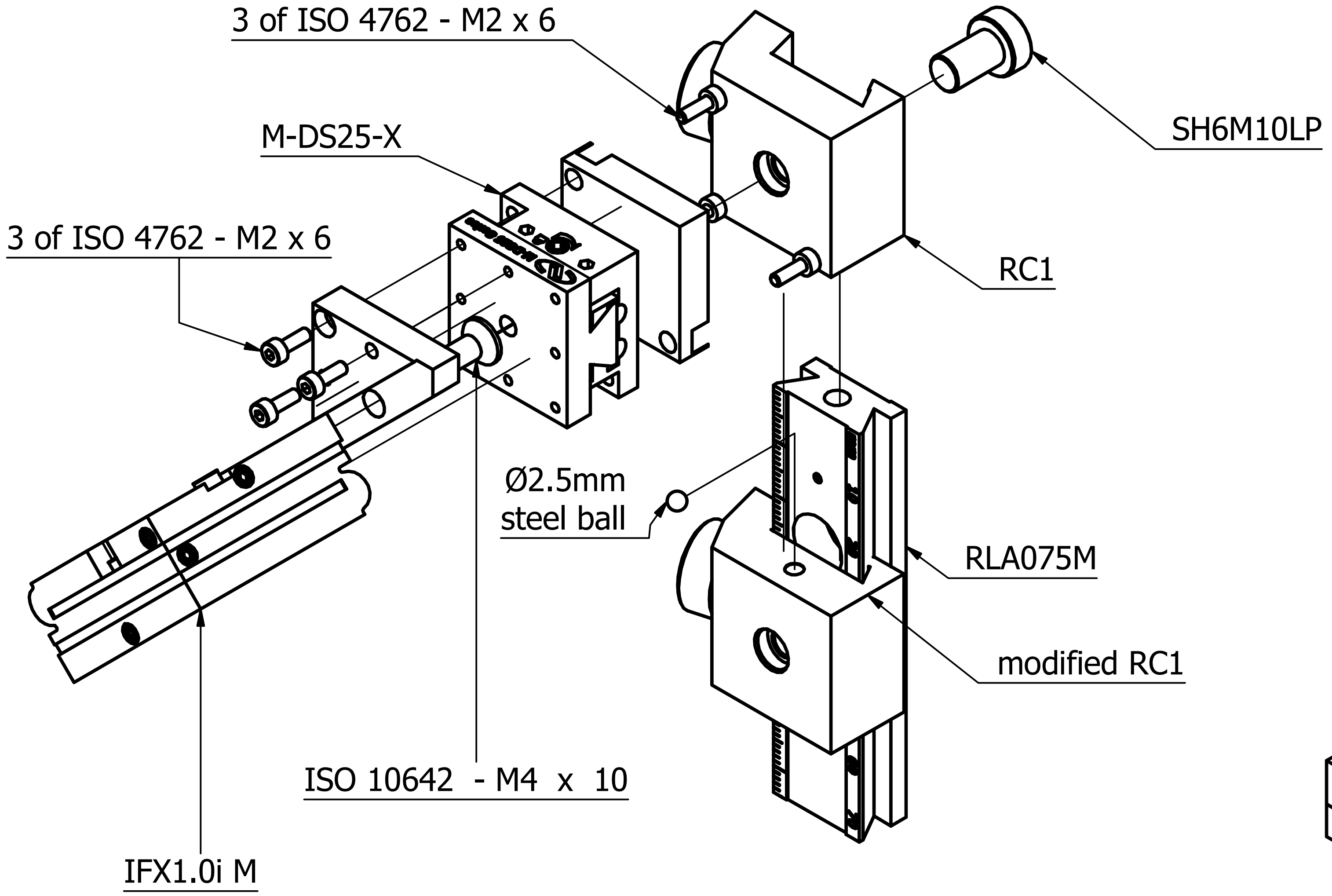

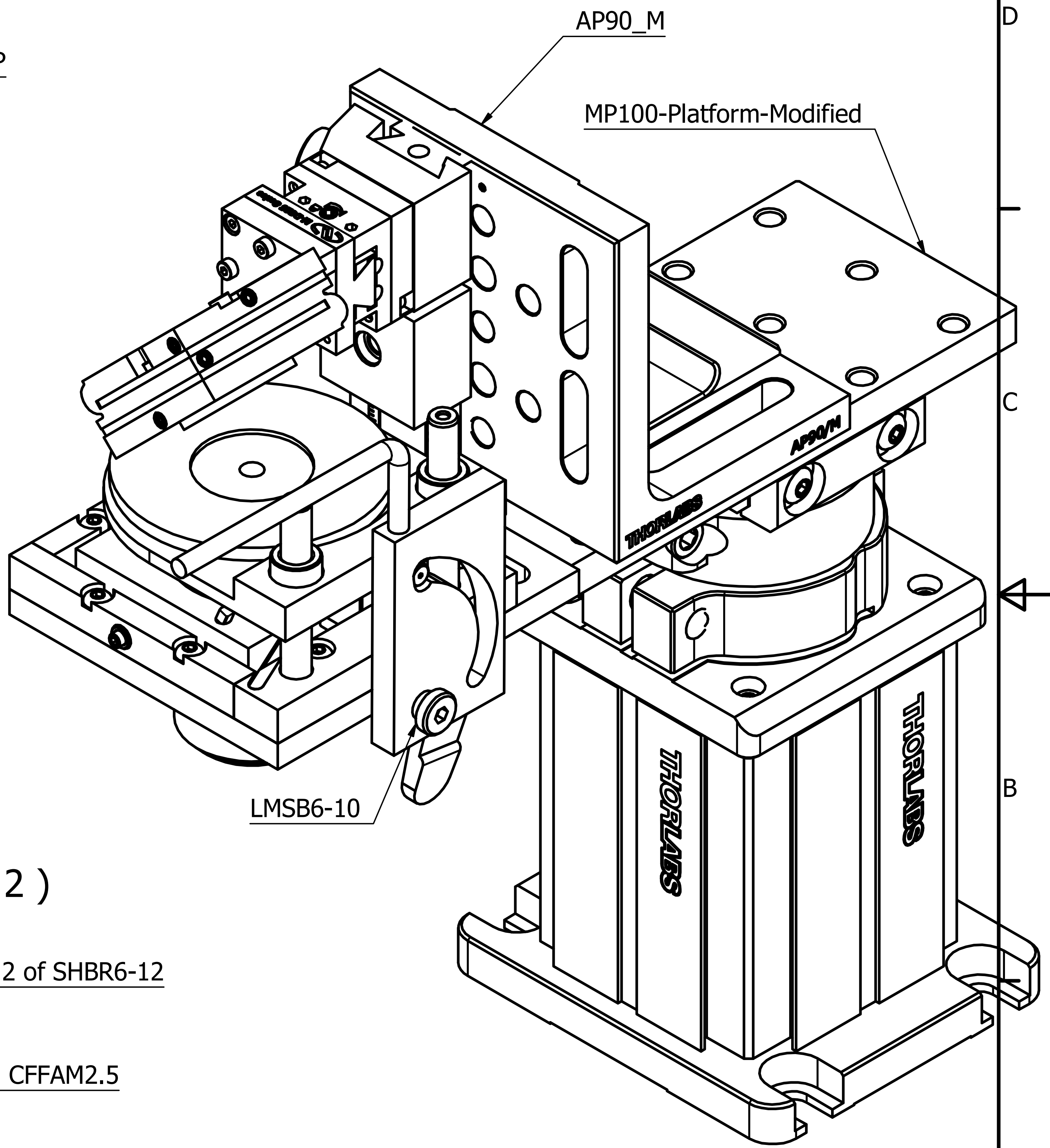

objective mount (1 : 2)

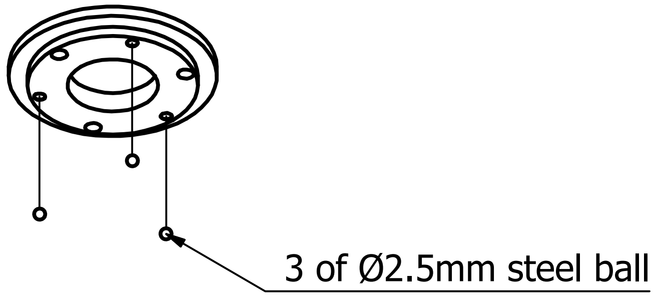

lifter plate (1 : 2)

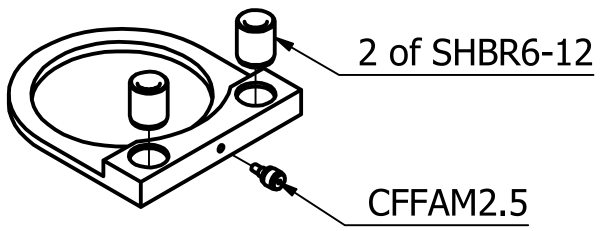

DESIGNED BY	CHECKED BY	APPROVED BY	DATE		DATE
map					13/05/2019

	ISSUE	SHEET
B24 Objective mount	2	1 / 12

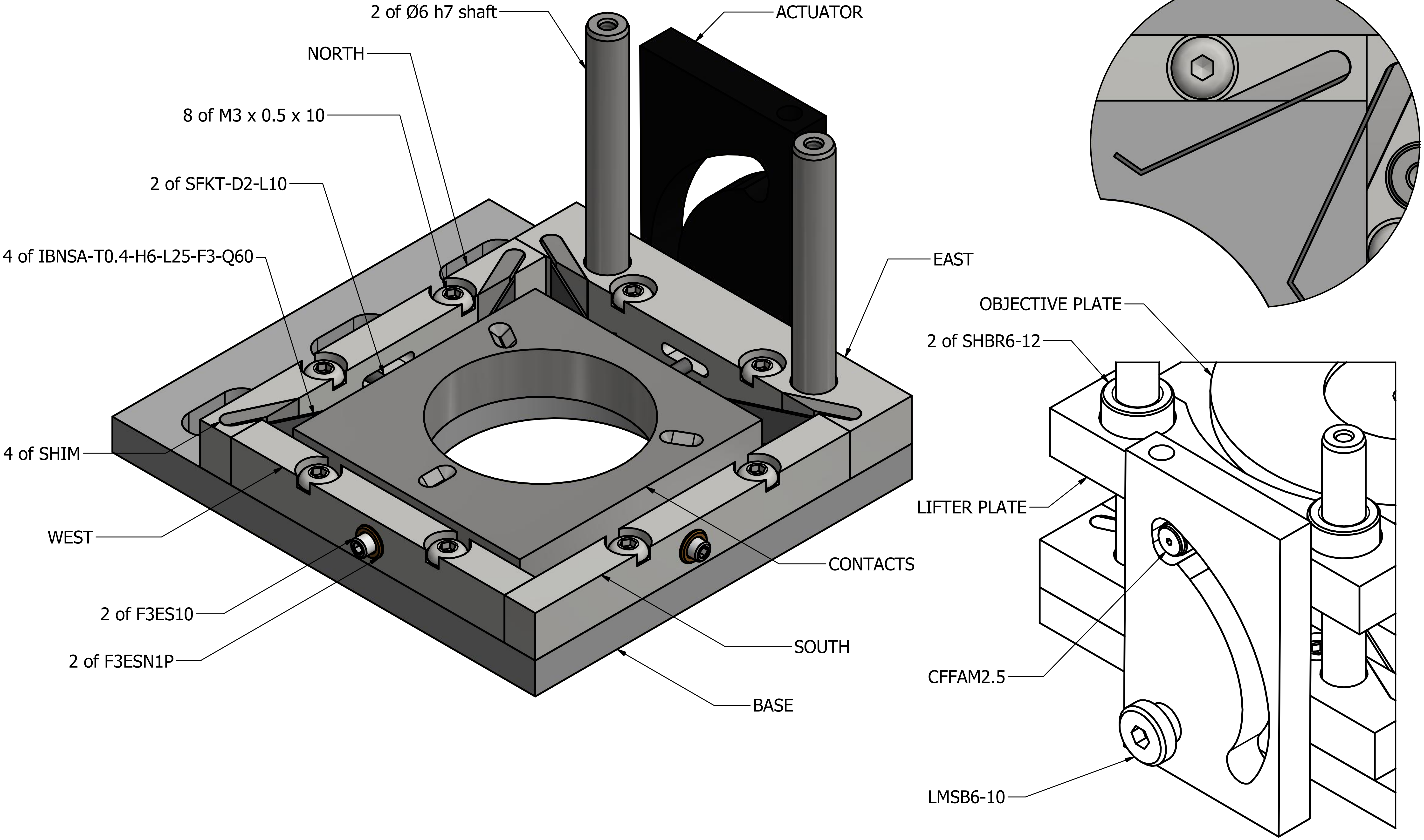

Designed by	Checked by	Approved by	Date	Date
map				13/05/2019

	Edition	Sheet
B24 Objective mount	2	2 / 12

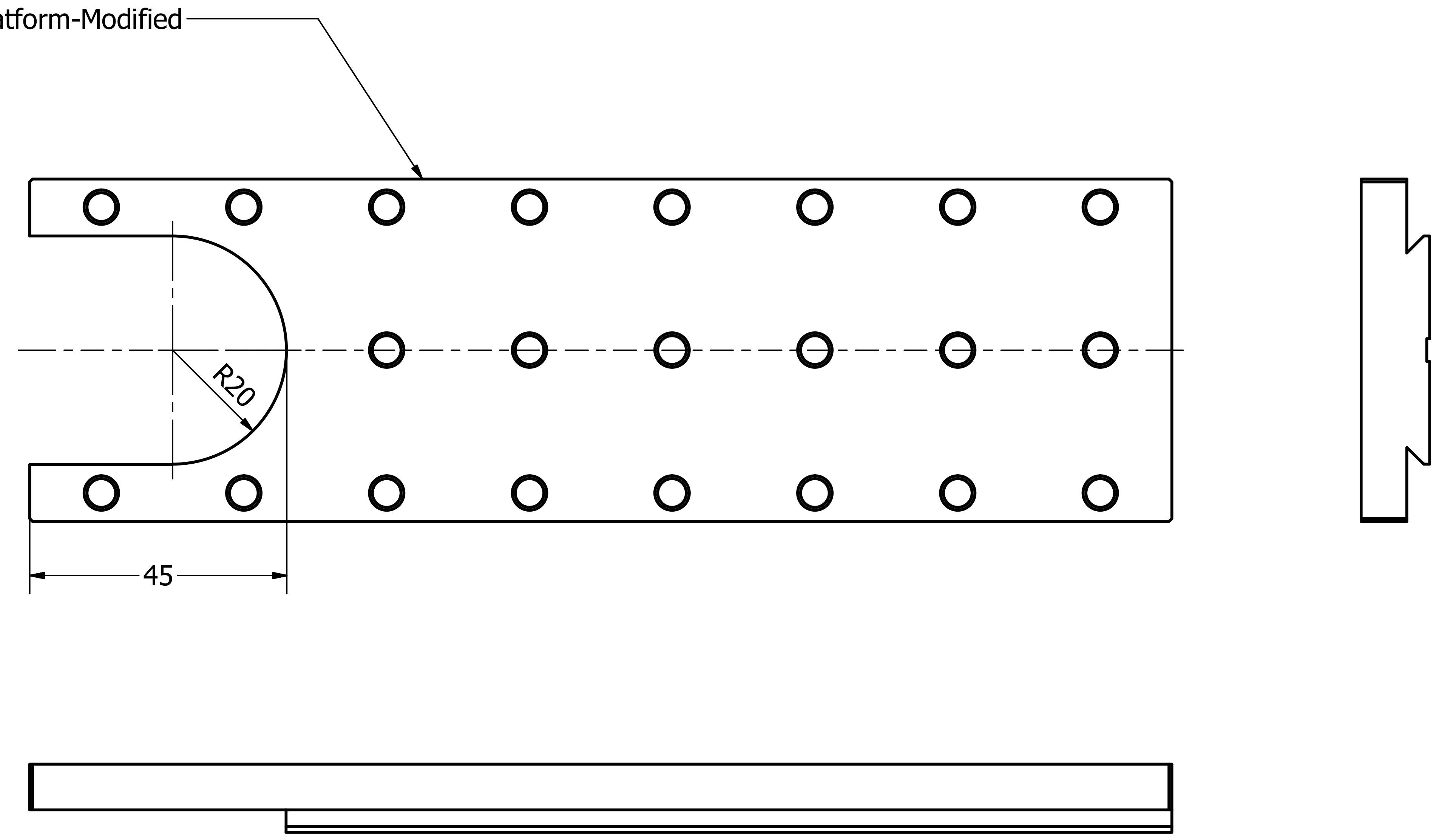

1 feature to be cut/milled.
Part supplied in aluminium.

Designed by	Checked by	Approved by	Date	Date
map				13/05/2019

B24 Objective mount	Edition 2	Sheet 3 / 12

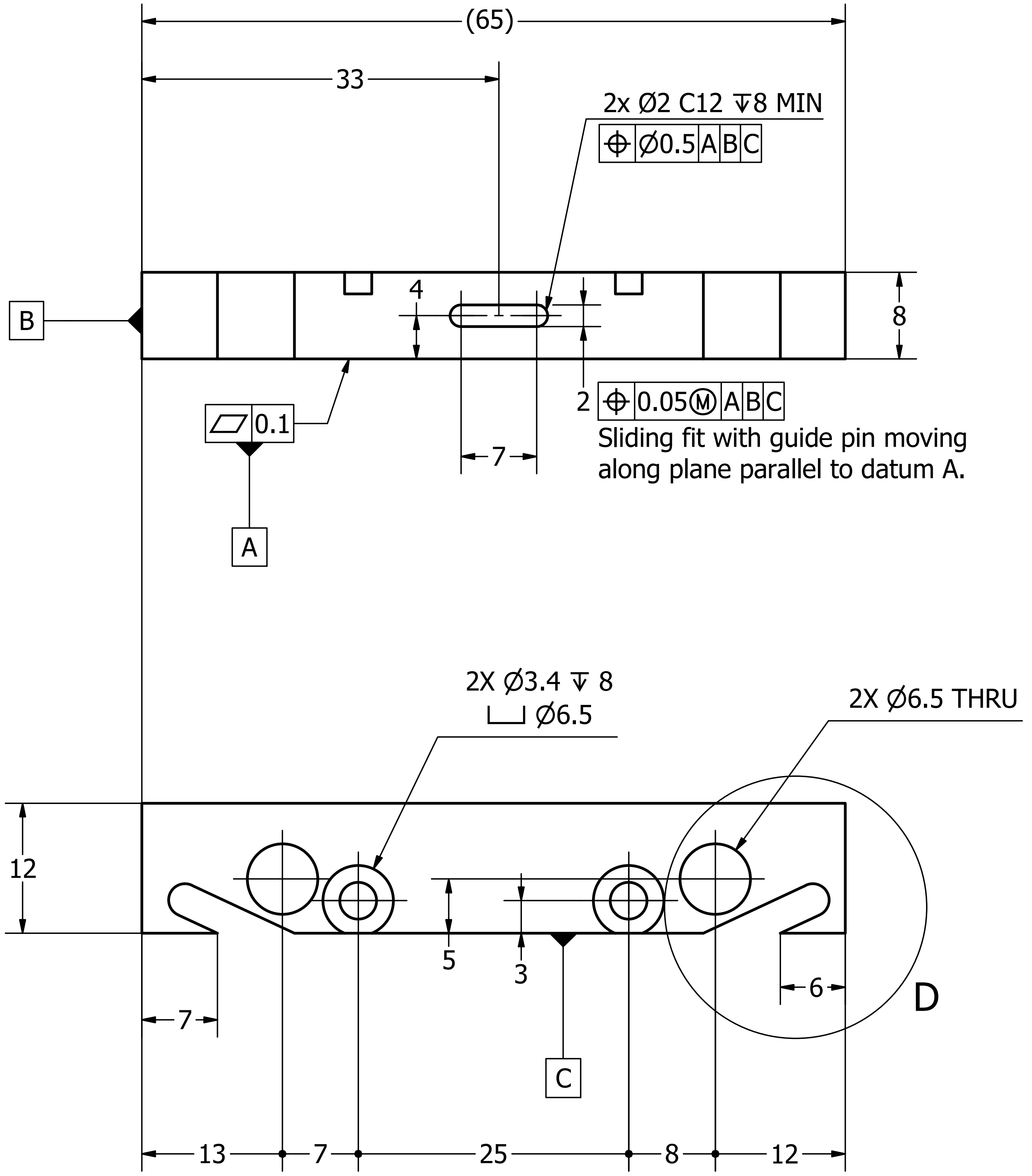

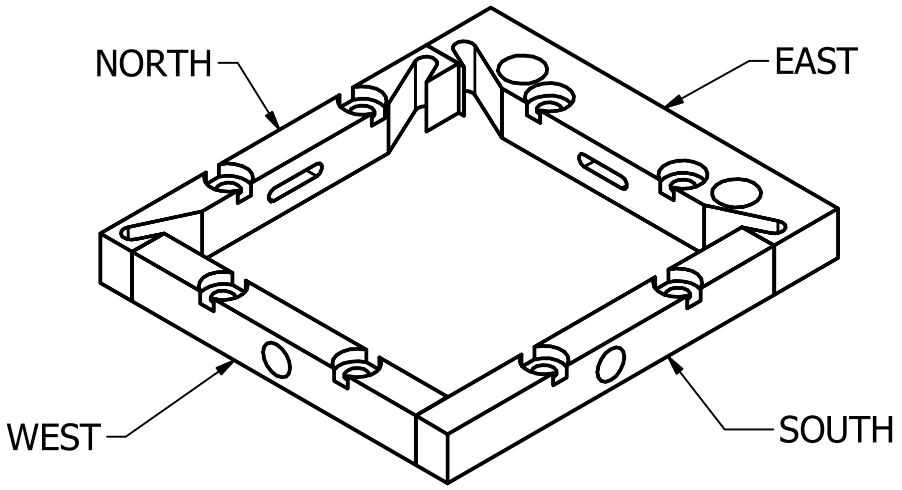

D (4 : 1)

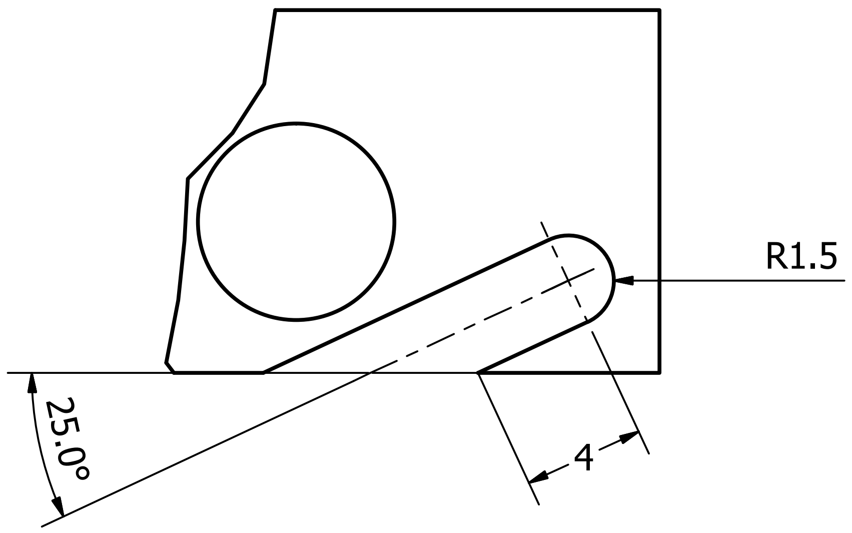

EAST (2 : 1)
1 off, steel
S275

Designed by	Checked by	Approved by	Date	Date
map				13/05/2019

B24 Objective mount	Edition 2	Sheet 4 / 12

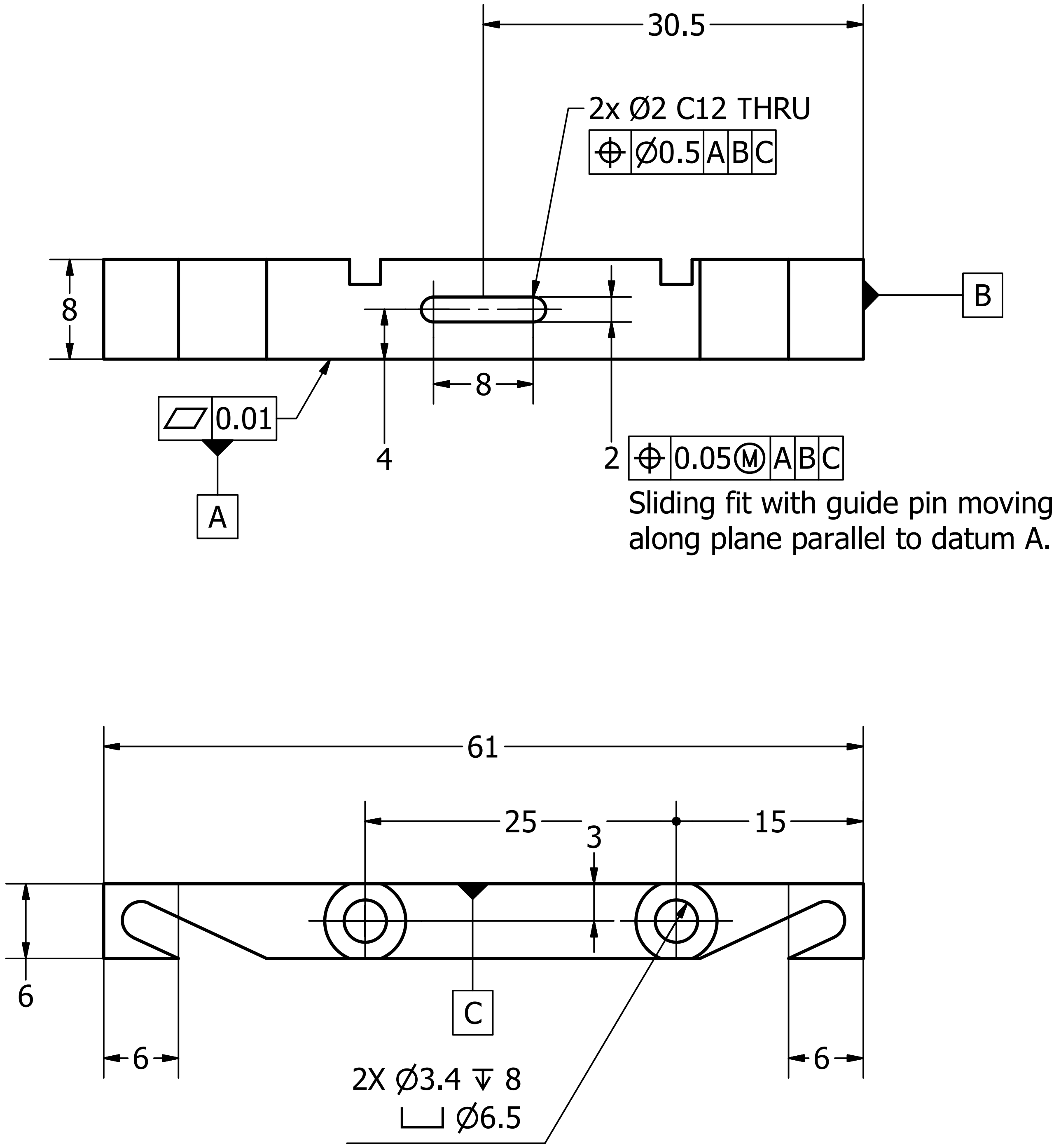

NORTH (2 : 1)
1 off, steel
S275

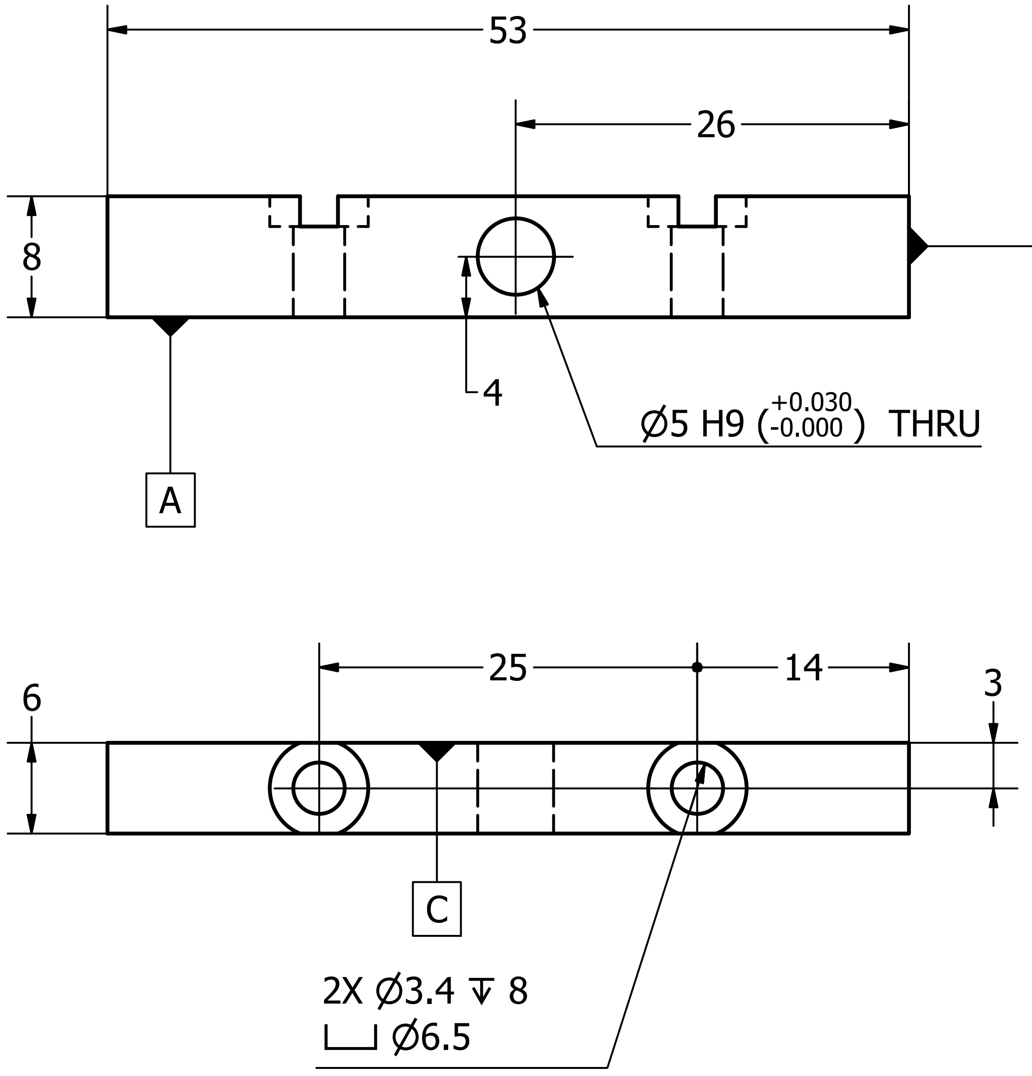

WEST (2 : 1)
1 off, steel
S275

Designed by	Checked by	Approved by	Date	Date	
map				13/05/2019	
		B24 Objective mount		Edition 2	Sheet 5 / 12

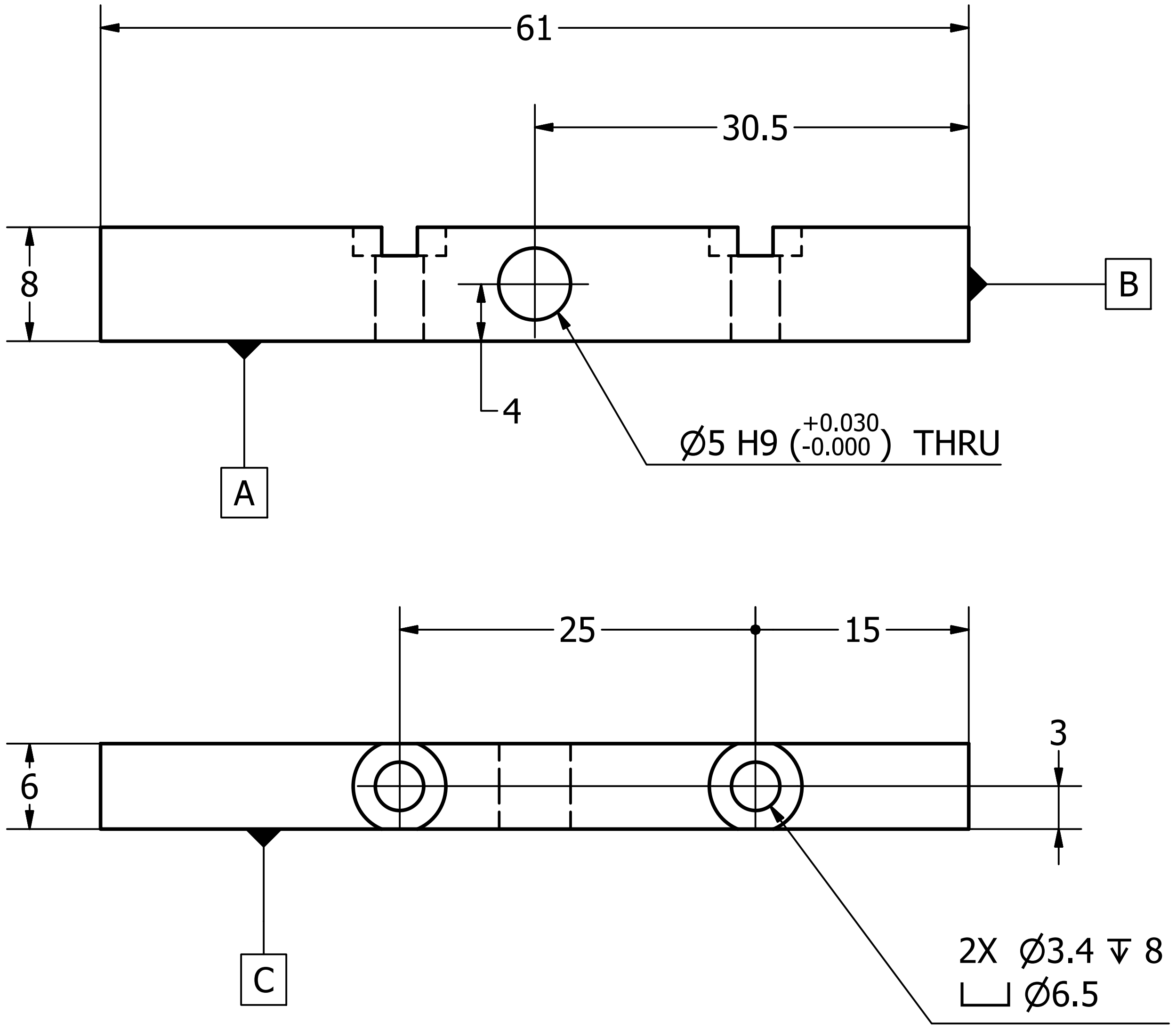

SOUTH (2 : 1)
1 off, steel
S275

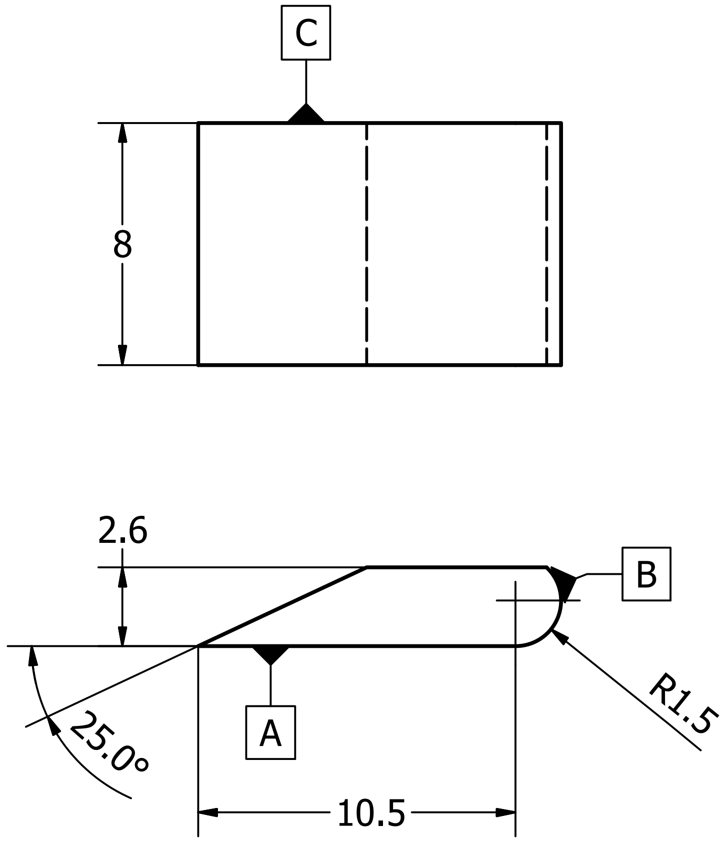

SHIM (4 : 1)
4 off, steel

Designed by	Checked by	Approved by	Date	Date
map				13/05/2019

	B24 Objective mount	Edition 2	Sheet 6 / 12

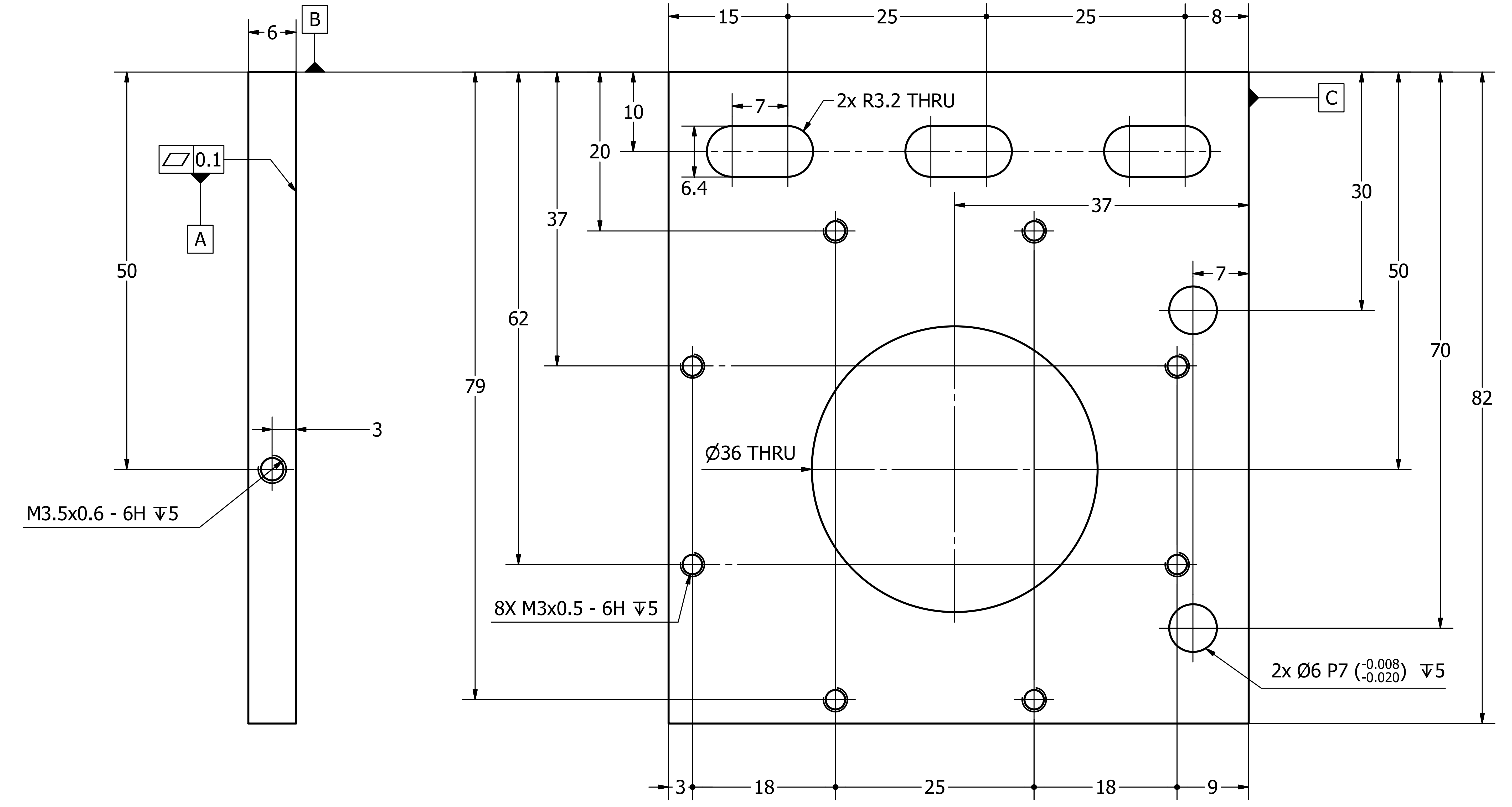

BASE (2 : 1)
1 off, steel
EN1A S275 or 6mm gauge plate

Designed by	Checked by	Approved by	Date	Date
map				13/05/2019

	Edition	Sheet
B24 Objective mount	2	7 / 12

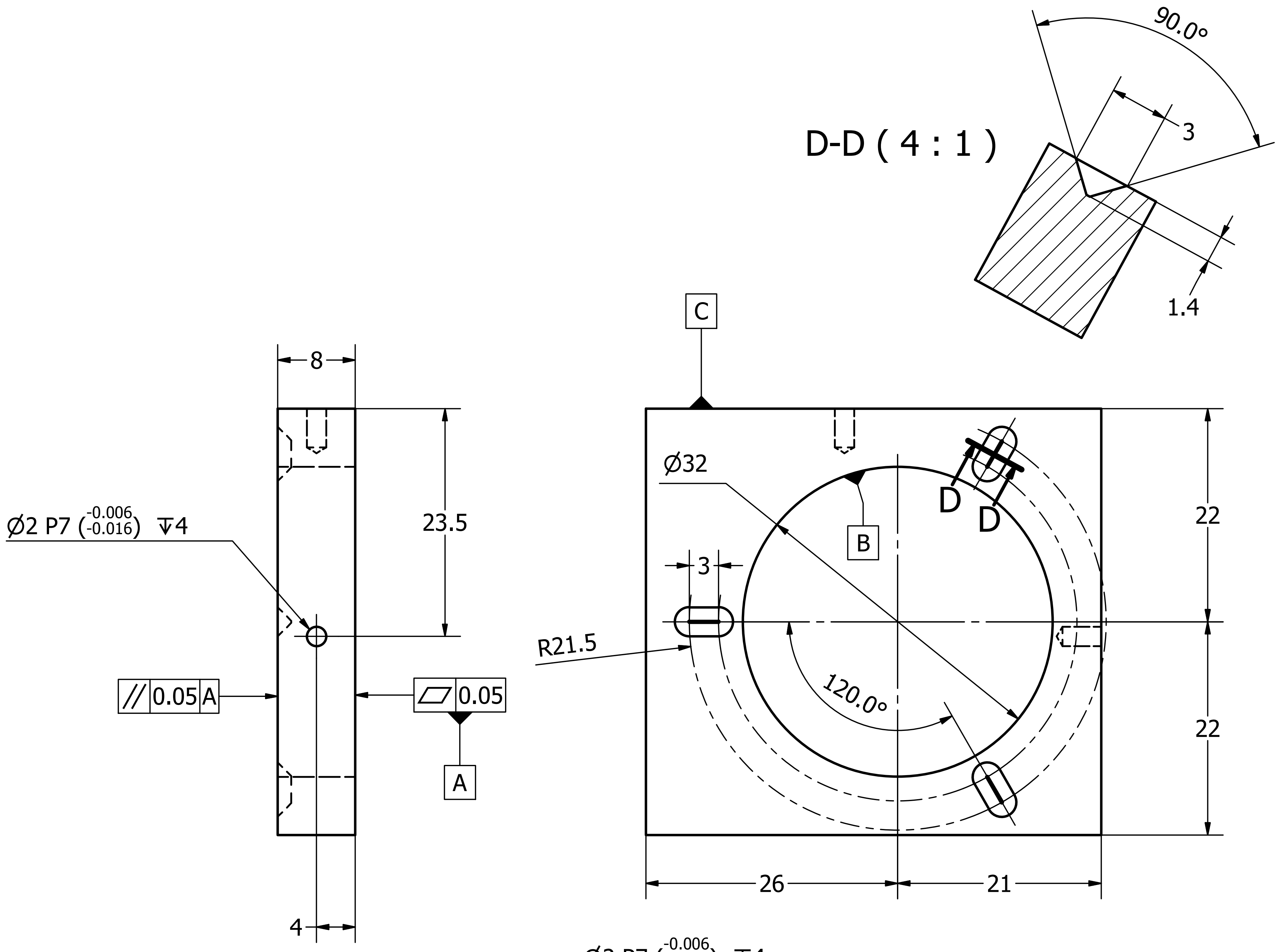

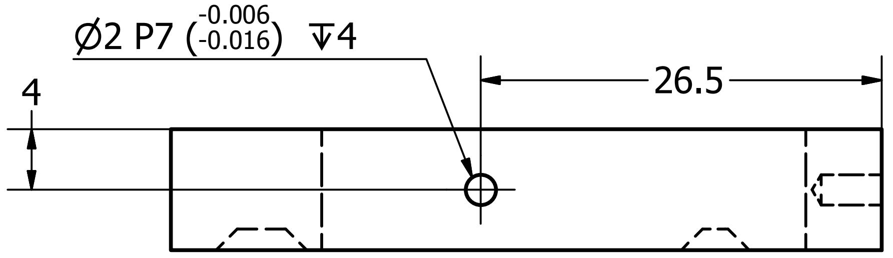

CONTACTS (2 : 1)

1 off, steel

S275 or 8mm gauge plate

Designed by	Checked by	Approved by	Date	Date
map				13/05/2019

B24 Objective mount	Edition 2	Sheet 8 / 12

CAM CL (centreline) co-ordinates along directions
AB and CB, with axis of feature D at (0,0).
(Supplied as CAM.CSV with 0.1 steps along AB.)

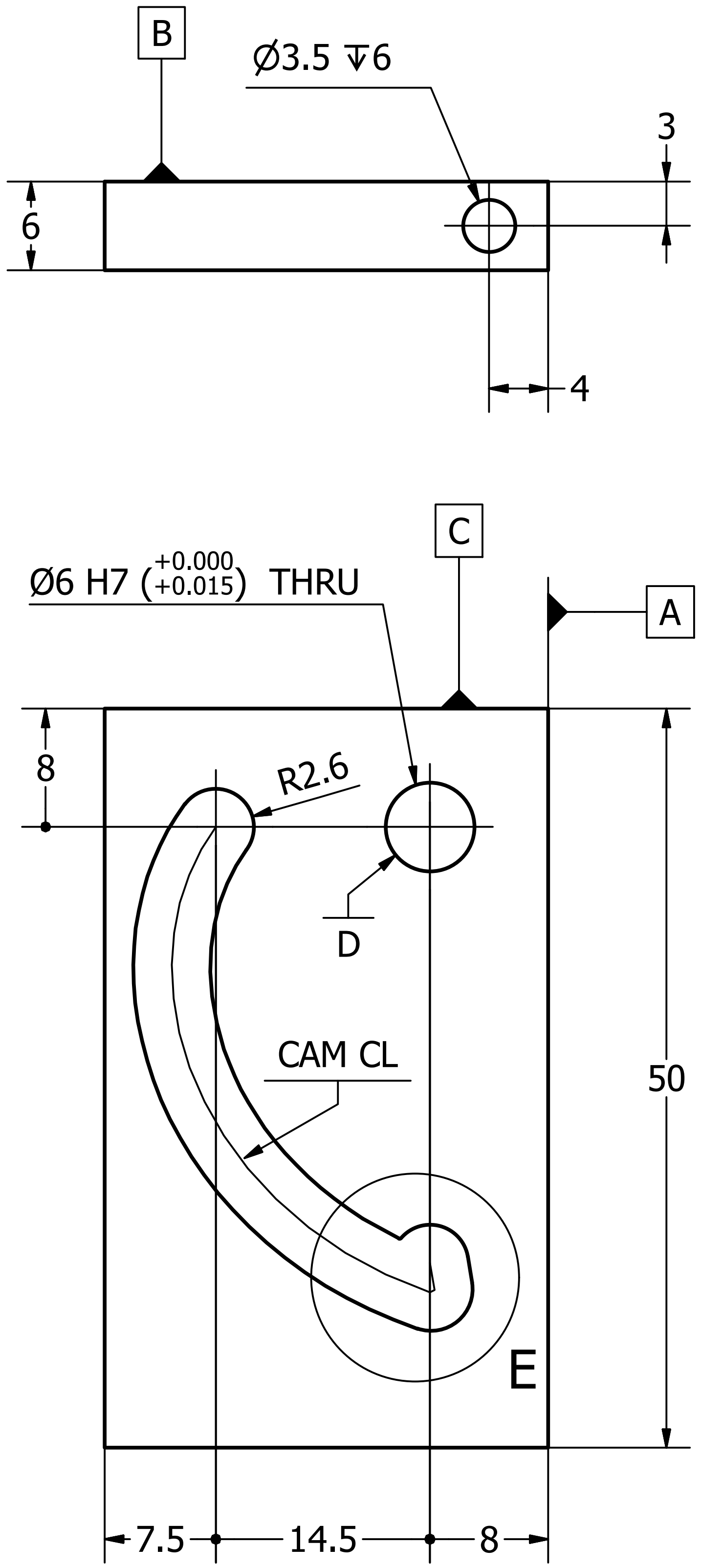

CAM CL		CAM CL	
AB	CB	AB	CB
0.0	14.5	14.0	16.9
0.5	14.9	14.5	16.8
1.0	15.2	15.0	16.7
1.5	15.5	15.5	16.5
2.0	15.7	16.0	16.4
2.5	16.0	16.5	16.2
3.0	16.2	17.0	16.0
3.5	16.4	17.5	15.8
4.0	16.6	18.0	15.6
4.5	16.8	18.5	15.3
5.0	16.9	19.0	15.1
5.5	17.0	19.5	14.8
6.0	17.1	20.0	14.5
6.5	17.2	20.5	14.2
7.0	17.3	21.0	13.9
7.5	17.4	21.5	13.6
8.0	17.4	22.0	13.2
8.5	17.5	22.5	12.8
9.0	17.5	23.0	12.4
9.5	17.5	23.5	12.0
10.0	17.5	24.0	11.6
10.5	17.5	24.5	11.1
11.0	17.4	25.0	10.6
11.5	17.4	25.5	10.1
12.0	17.3	26.0	9.5
12.5	17.2	26.5	9.0
13.0	17.2	27.0	8.3
13.5	17.1	27.5	7.7

E (10 : 1)

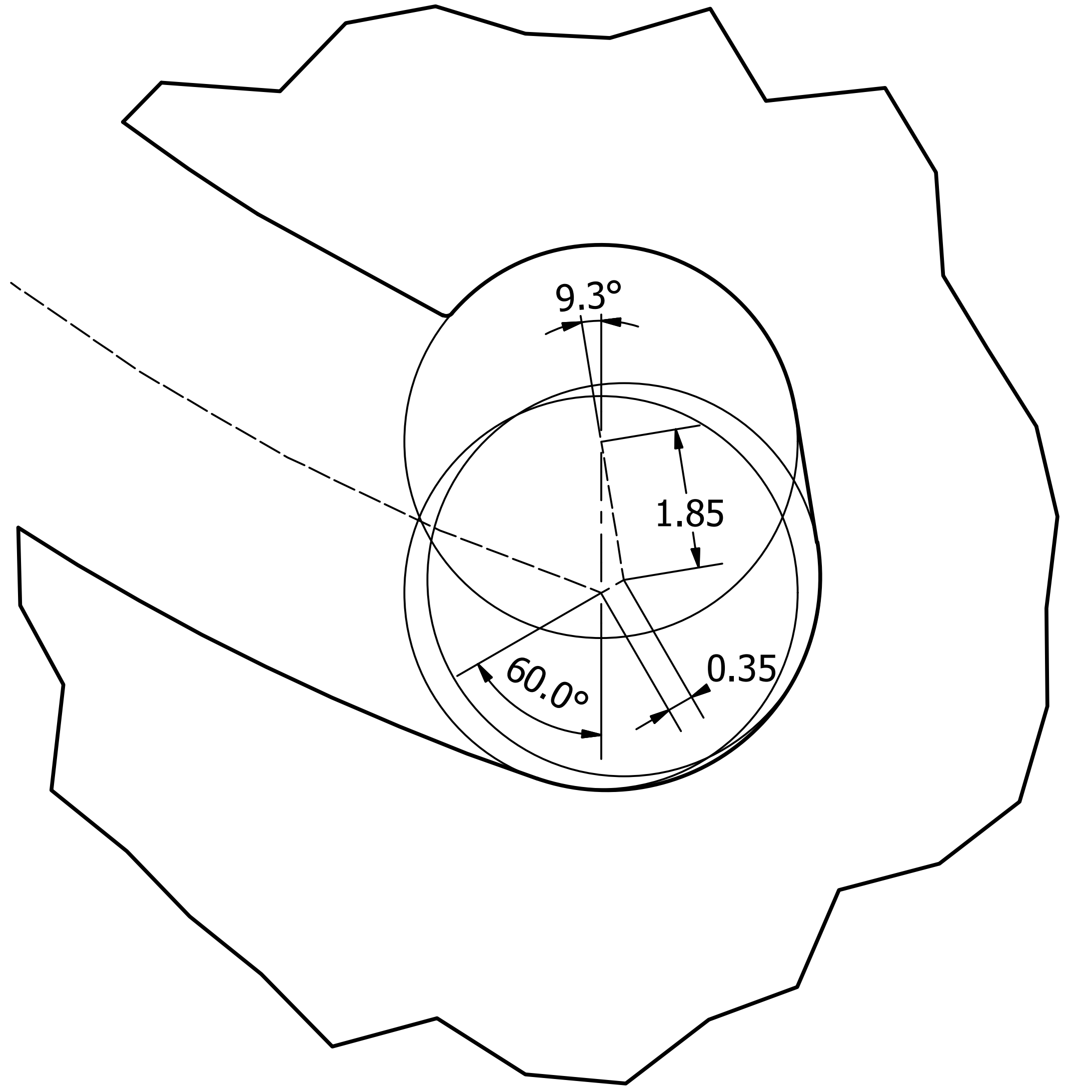

ACTUATOR (2 : 1)
1 off, aluminium

Designed by	Checked by	Approved by	Date	Date
map				13/05/2019

B24 Objective mount

Edition 2

Sheet 9 / 12

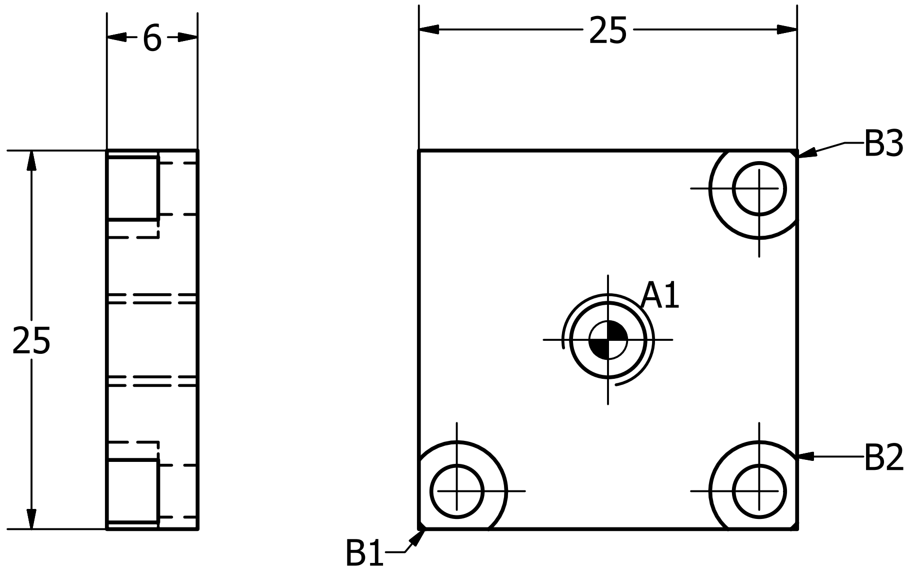

HOLE TABLE			
HOLE	XDIM	YDIM	DESCRIPTION
A1	0	0	M6x1 - 6H
B1	-10	-10	Ø3,4 THRU<br>⌴ Ø6,5 ↧ 3,4
B2	10	-10	
B3	10	10	

drive to dovetail adapter (2 : 1)
1 off
Aluminium

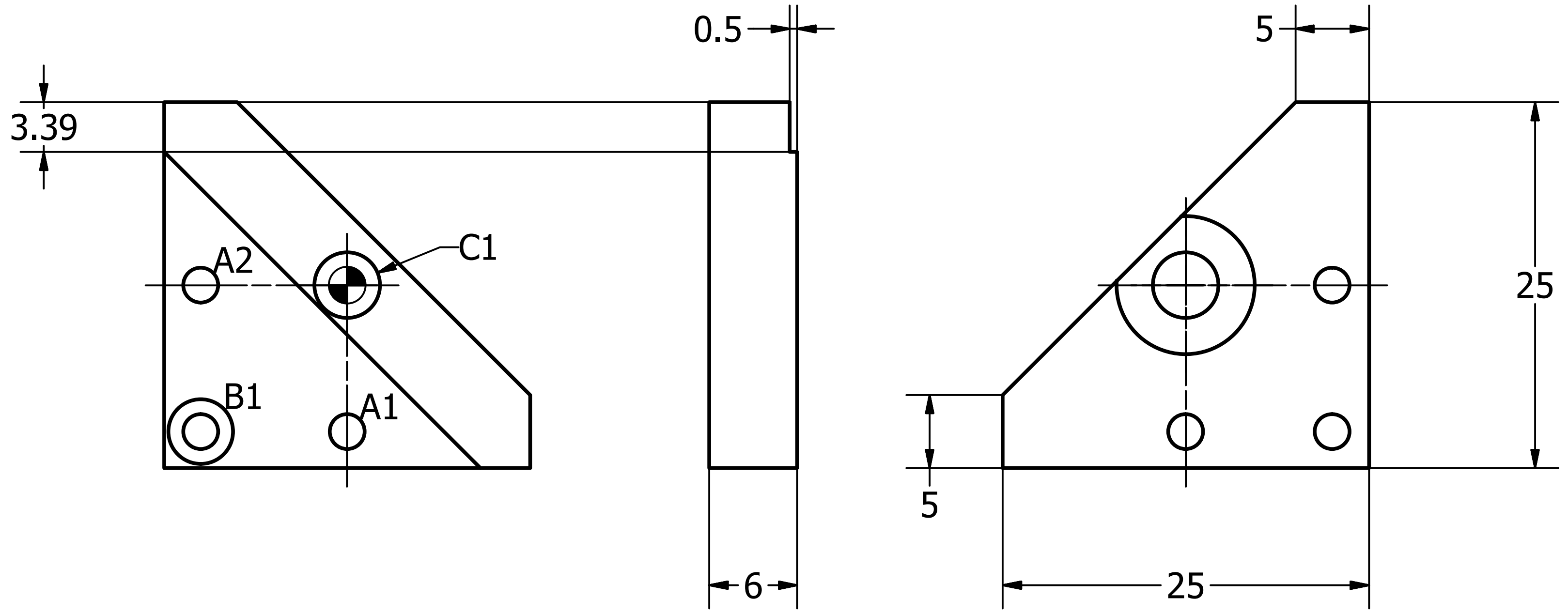

HOLE TABLE			
HOLE	XDIM	YDIM	DESCRIPTION
A1	0	-10	Ø2,4 THRU
A2	-10	0	
B1	-10	-10	Ø2,4 THRU<br>⌴ Ø4,4 ↧ 2,3
C1	0	0	Ø4,5 -8 DEEP<br>⌵ Ø9,4 X 90°

mirror to drive adapter (2 : 1)
1 off
Aluminium

ALUMINIUM
linear dimensions in mm

DESIGNED BY	CHECKED BY	APPROVED BY	DATE	DATE
map				13/05/2019

	ISSUE	SHEET
B24 Objective mount	2	10 / 12

lifter plate (2 : 1)
1 off
Aluminium

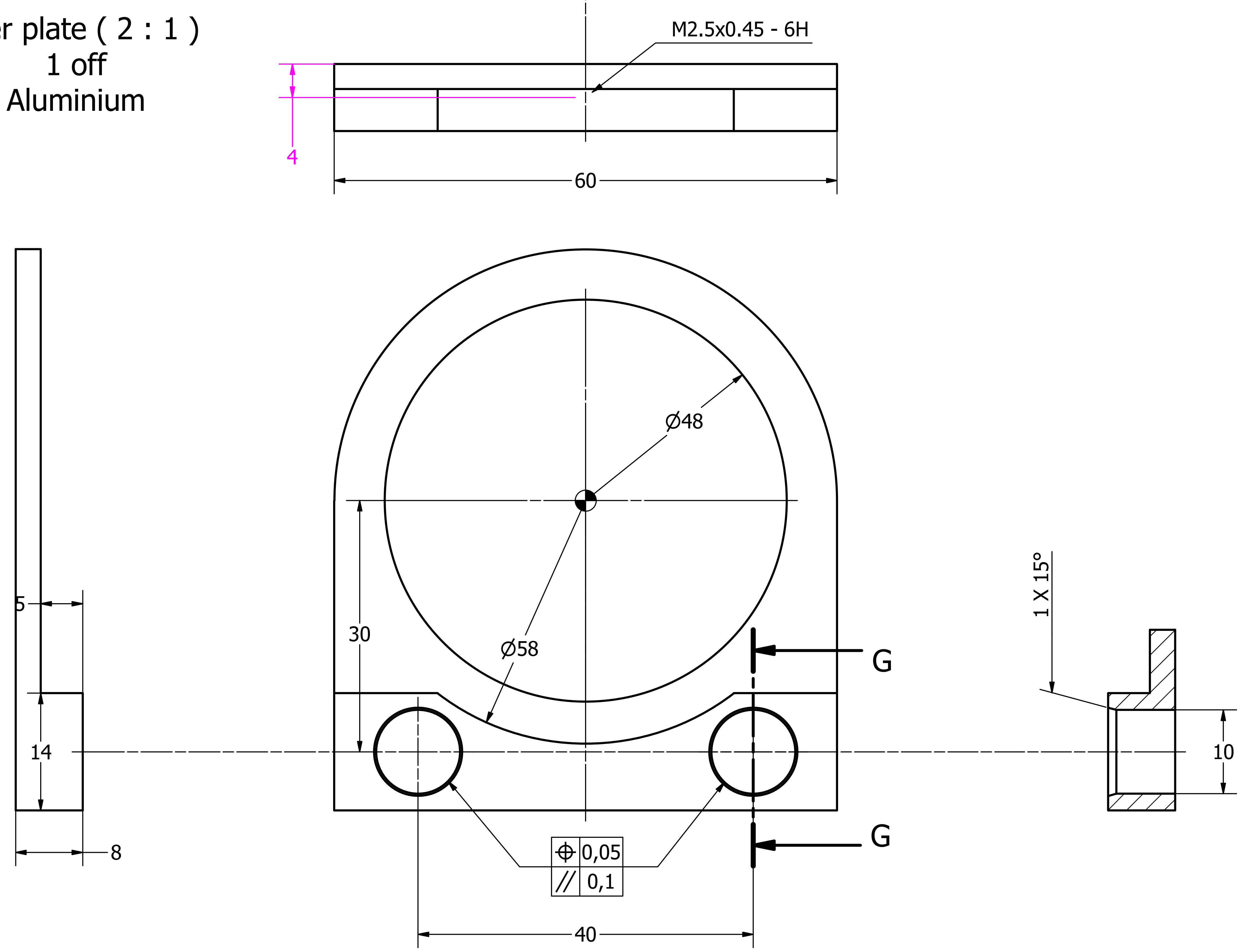

ALUMINIUM

linear dimensions in mm

DESIGNED BY	CHECKED BY	APPROVED BY	DATE	DATE
map				13/05/2019

	ISSUE	SHEET
B24 Objective mount	2	11 / 12

objective plate (2 : 1)
1 off
Aluminium

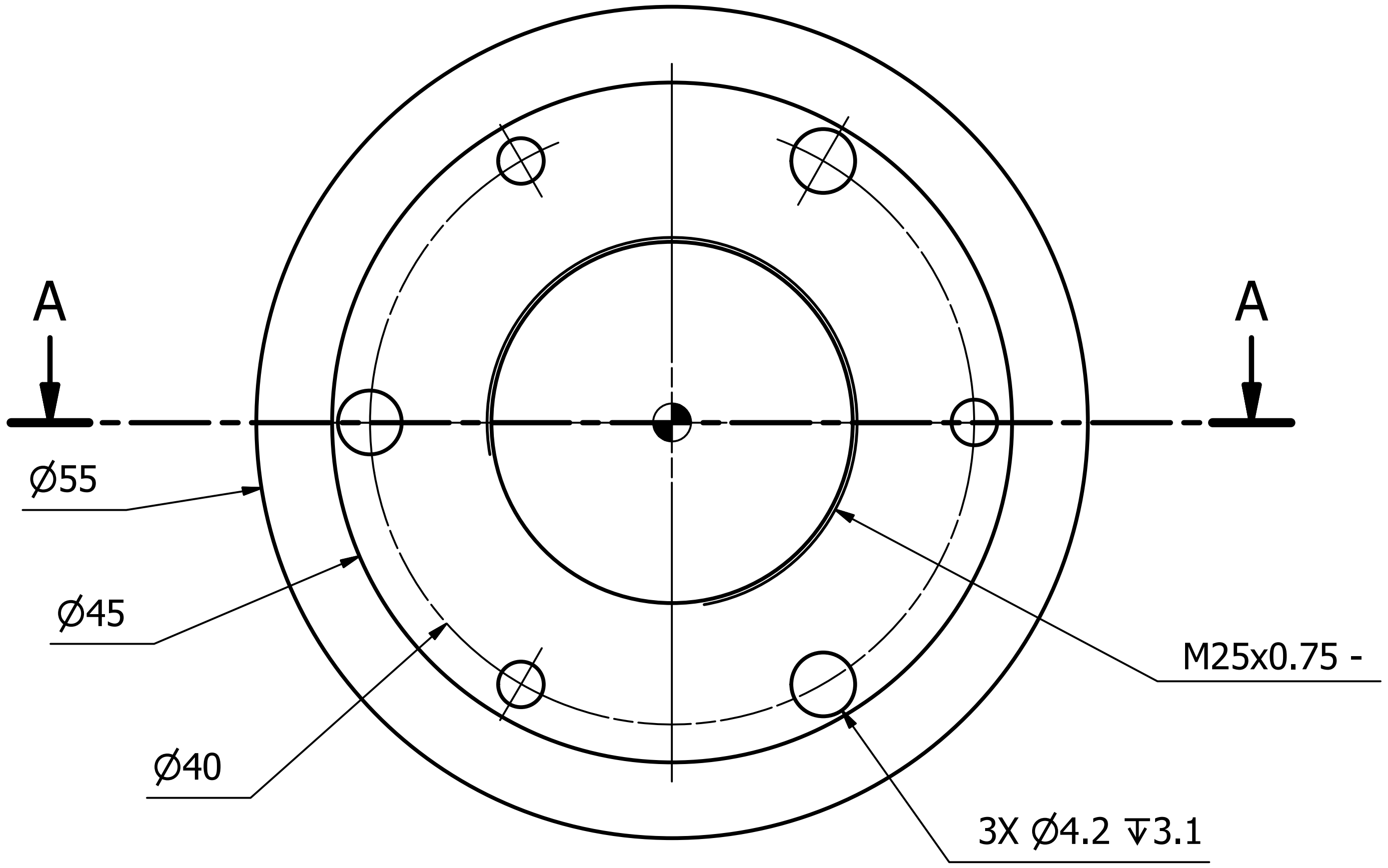

A-A (2 : 1)

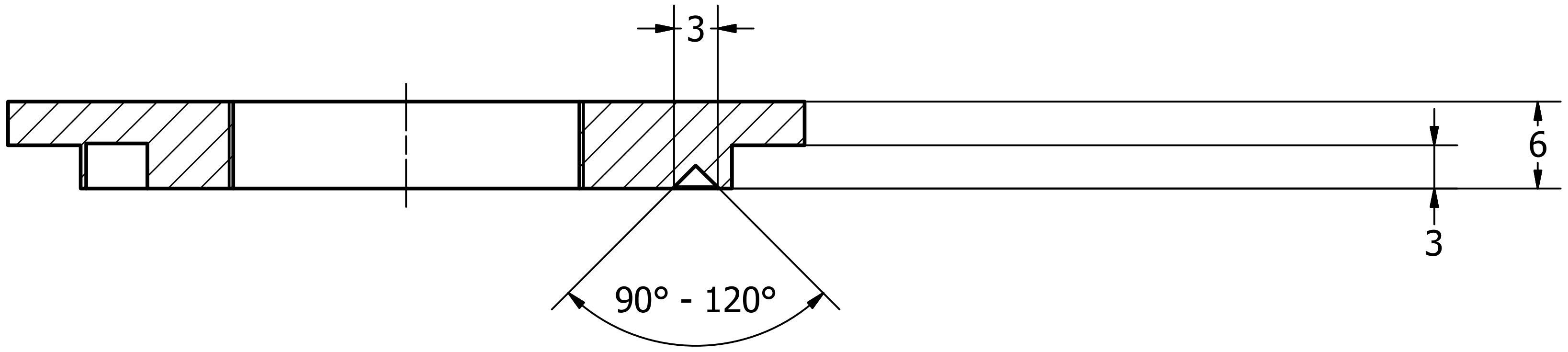

modified RC1 (2 : 1)
1 part modification
Part supplied in aluminium

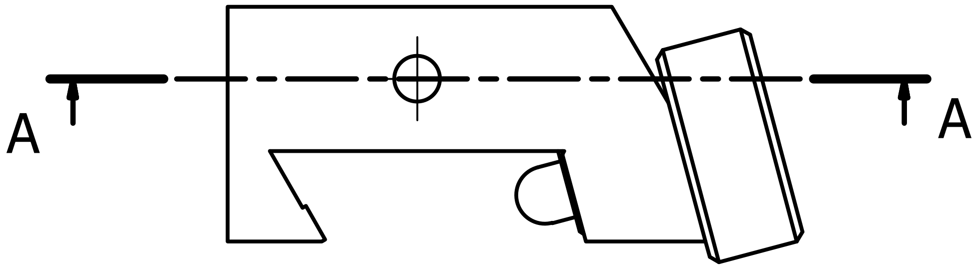

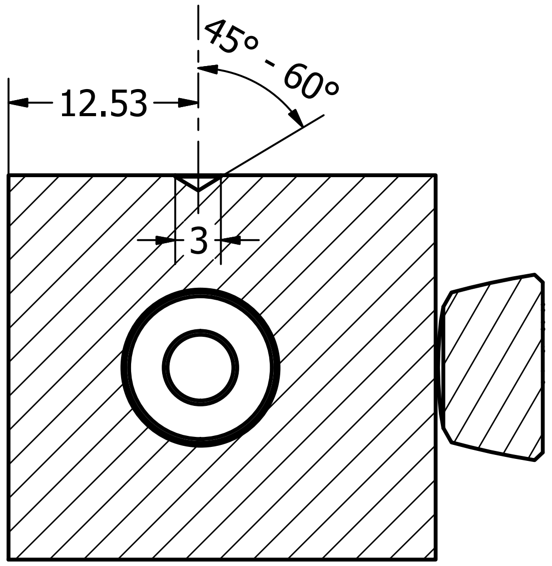

A-A (2 : 1)

ALUMINIUM
linear dimensions in mm

DESIGNED BY	CHECKED BY	APPROVED BY	DATE	DATE
map				13/05/2019

	ISSUE	SHEET
B24 Objective mount	2	12 / 12
