# Supplementary material for: CryoSIM: super-resolution 3D structured illumination cryogenic fluorescence microscopy for correlated ultrastructural imaging
Source: Optica. 2020 Jul 13;7(7):802–12. doi: 10.1364/OPTICA.393203 (PMC8262592; doi:10.1364/OPTICA.393203)
Supplement: Supplementary file 2 [file optica-7-7-802-d001.zip › Plate.PiezoToLeadscrew.pdf]

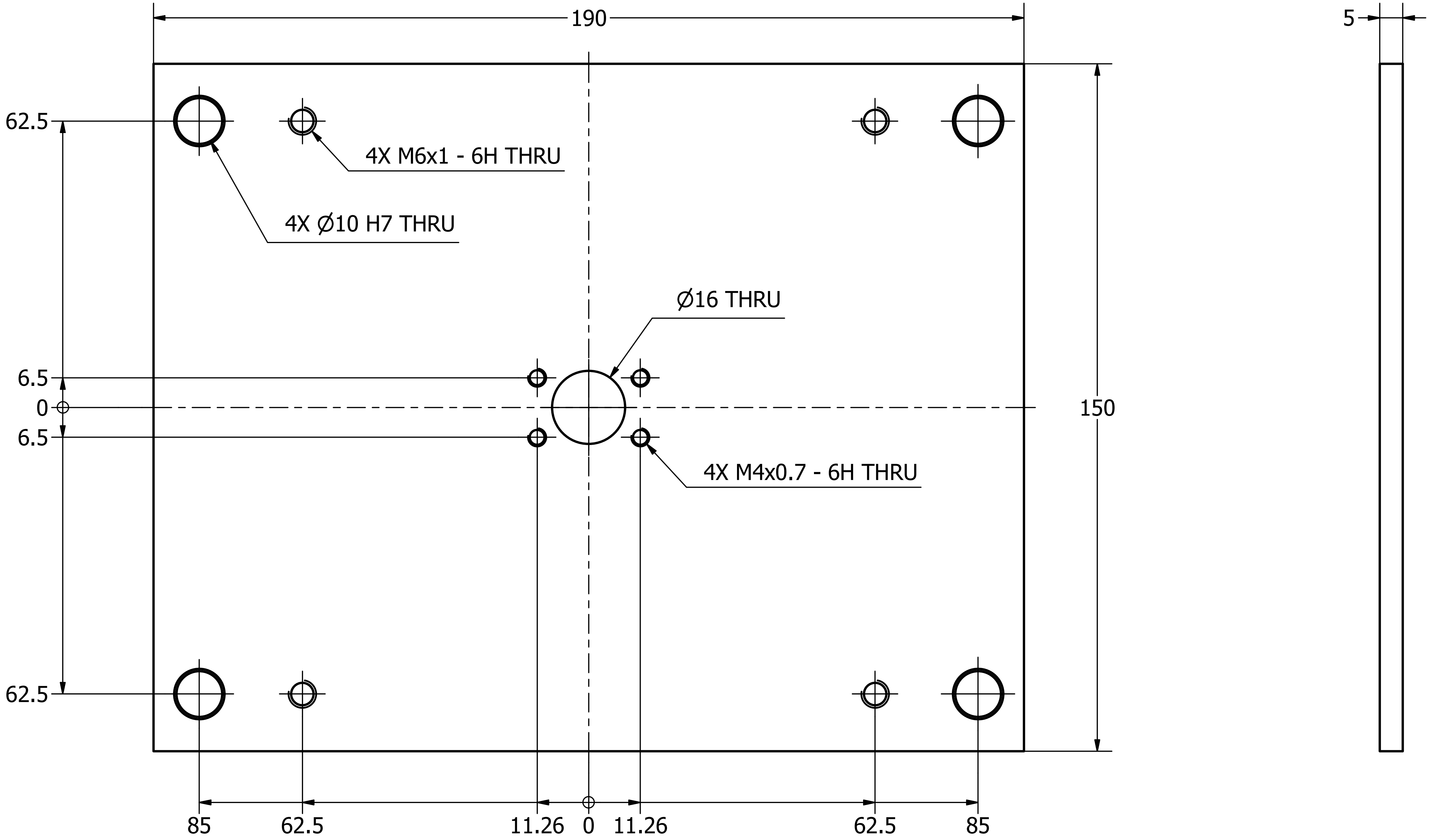

1 OFF, ALUMINIUM

Designed by	Checked by	Approved by	Date		Date	
map					14/08/2018	

3rd ANGLE PROJECTION	Micron Oxford	
		Plate.PiezoToLeadscrew
Edition		
Sheet	1 / 1	
