# Supplementary figures and images for: CryoSIM: super-resolution 3D structured illumination cryogenic fluorescence microscopy for correlated ultrastructural imaging
Source: Optica. 2020 Jul 13;7(7):802–12. doi: 10.1364/OPTICA.393203 (PMC8262592; doi:10.1364/OPTICA.393203)

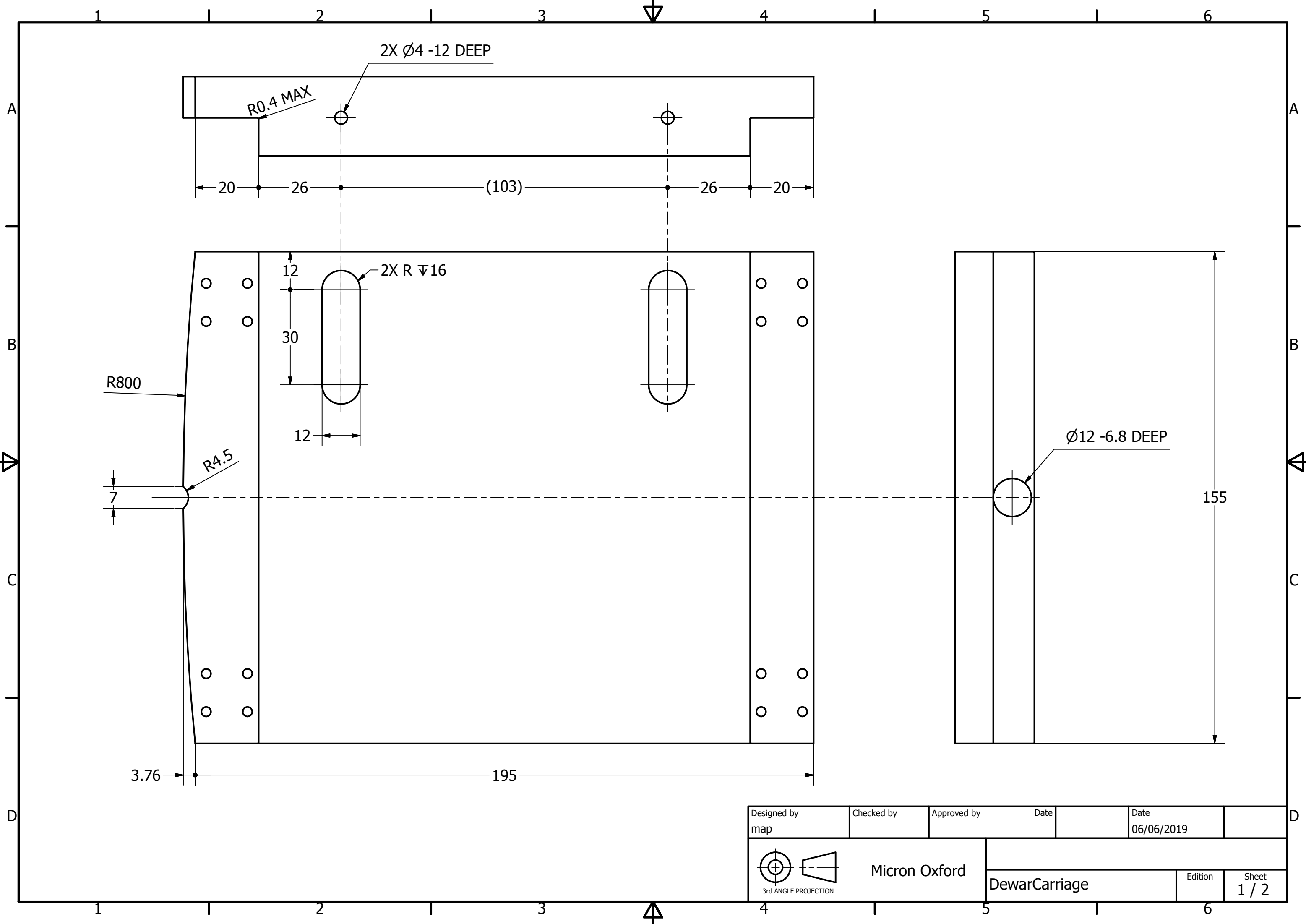

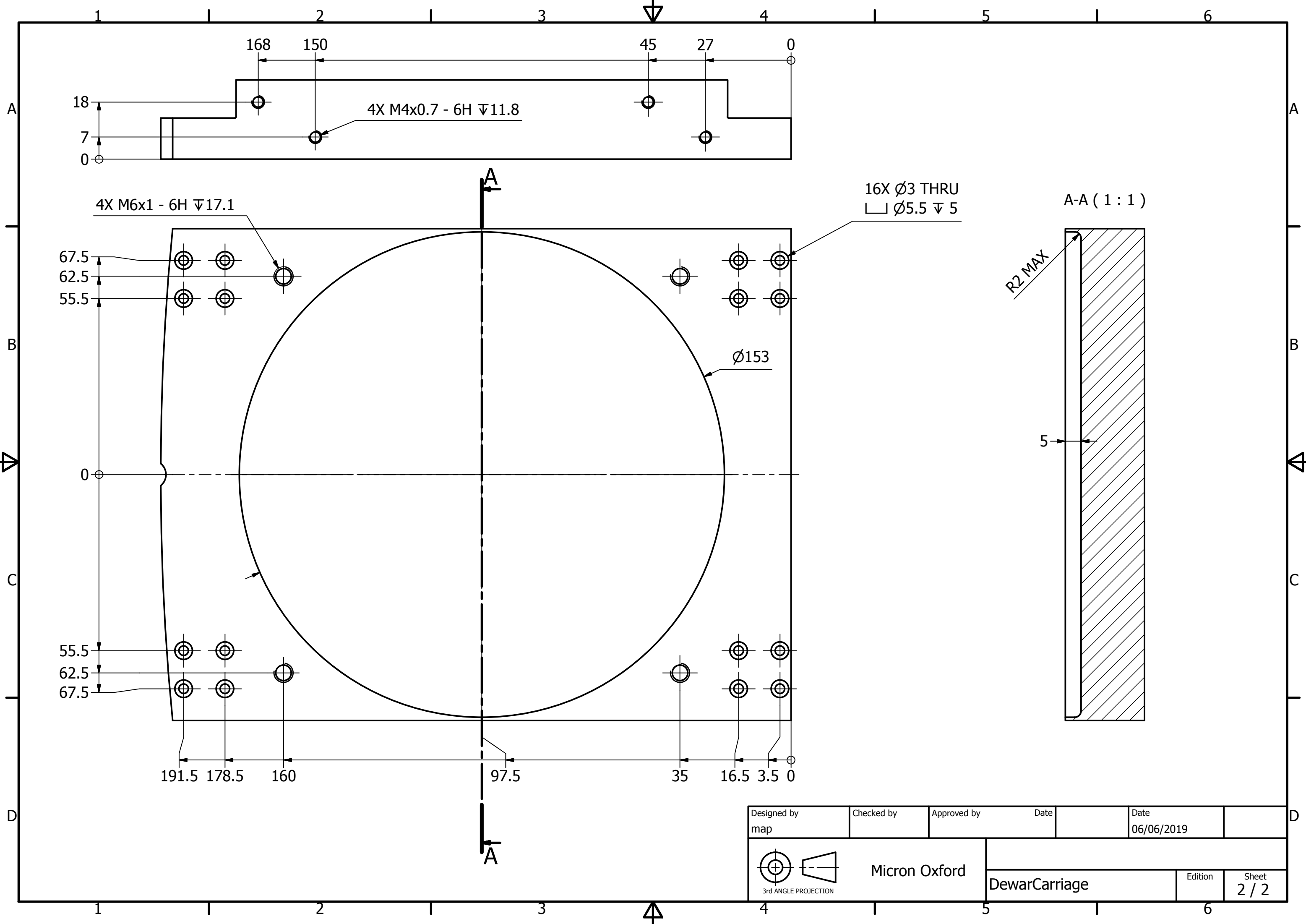

|                                                                                                               |            |             |                |            |
|---------------------------------------------------------------------------------------------------------------|------------|-------------|----------------|------------|
| Designed by<br>map                                                                                            | Checked by | Approved by | Date           | 06/06/2019 |
| 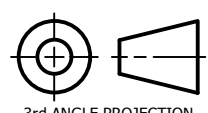<br>3rd ANGLE PROJECTION |            |             | Edition        |            |
|                                                                                                               |            |             | Sheet<br>2 / 2 |            |

Supplement: Supplementary file 2 [file optica-7-7-802-d001.zip › DewarCarriage.pdf]
